# A Report on 2527 Cases of Primary Suture

**Published:** 1918-09

**Authors:** 


**A Report on 2537 Cases of Primary Suture of War Wounds,** By René Lemaitre. *Lyon Chirurgical*, Jan. and Feb. 1918.

This report is based on 29 months experience with the control of end results. Lemaitre operated before the seventh hour in 295 cases; in 1862 cases between the seventh and fifteenth hours, and in 126 cases after 15 hours. In all he did 2537 primary sutures, 209 delayed primary sutures and 307 early secondary sutures. In the early period of his experience he did primary sutures in only 44 o/o of the cases — now he does it in 79 o/o.

He does not do a primary suture under the following conditions;

1. When the wounds are slight and not likely to be infected; as, for example, small fragment wounds of face and hands without injury to bone tendon or joints. As these missiles have not passed through clothes they are not likely to lead to infection.

2. Punctiform, transfixing wounds due to rifle balls without bone, joint, vascular, or nerve lesions.

3. When the general condition of the patient is bad and when the pulse is 120 or more.

4. When the time following the injury exceeds 24 hours. The earlier the operation of primary suture, the better the results.

5. Whenever there is a change in note about the wound, suggesting presence of gas as heard by the stethoscope. In these cases it is safer to do a delayed suture.

6. Never do a primary suture in a part distal to a ligated essential or terminal artery.

*Technic of Excision and Primary Suture.* Lemaitre removes all tissue dead and dying along the whole tract by making an incision around and close to the wound of entrance and enlarging it up and down or transversely as indicated. All foreign bodies are carefully searched for and removed as well as all loosened pieces of bone. Blood vessels are tied with fine catgut and only the vessel wall caught in the ligature. Nerve lesions are sutured at once, and if near a fracture the nerve suture is protected with muscles by canalization; otherwise this is not necessary. Most vascular lesions require double ligation; small lateral wounds may occasionally be sutured. Articular injuries are carefully cleaned and repaired, bone injuries are cleaned, loose pieces of bone or fragments removed, the periosteum is saved, sharp points are trimmed off, and the marrow adjacent to the fracture is scooped out and parts immobilized. Severe bony injury in articulations may require immediate resection. Muscles and aponeuroses are excised liberally along the tract of the wound even well into healthy tissue. Tendons, if cut, are sutured after freshening ends, occasionally anastomoses may be necessary. All contused hemorrhagic subcutaneous tissues should be excised. *In the excision of the wounds a curved scissors is the most useful instrument.* After making these excisions Lemaitre washes the wound with 5 o/o tincture of iodin which not only sterilizes the surface of the tissues but dries the cut surfaces. The wound is then drawn together with a small thread drain, as experience has shown that there is likely to be some discharge as the result of the use of the tincture of iodine. The drainage threads are removed in 3 or 4 days as a rule and the skin stitches in the middle of the second week. In some cases, owing to the presence of the above mentioned contraindications to primary suture, a delayed primary suture is to be done. Lemaitre makes a primary excision or débridement leaving the wound wide open covered with a dry dressing which remains in place 5 to 6 days. He makes no use of Dakin's solution as he has seen no benefit therefrom. At the dressing he washes the adjacent skin with oleate of sodium soap, dries with ether, and washes wound with tincture of iodin. If the bacteriological control (absence of streptococci Tissier) is satisfactory, a secondary closure of the wound is carried out. Experience has shown that neither those wounds which are closed originally or these that are closed secondarily are bacteriologically sterile. Lemaitre feels sure that he has done successful primary sutures in wounds containing streptococci.

Post-operative course of primary suture cases is usually very satisfactory. About 95 o/o heal without any trouble. Partial and complete failures occurring in the remaining. Pain, increase in pulse rate, rise in temperature, disturbance in general condition with local tenderness will usually indicate that there is infection in the closed wound.

